# Subarachnoid Neurocysticercosis Caused by Larval-Stage *Taenia crassiceps* Tapeworm, Slovenia

**DOI:** 10.3201/eid3109.250014

**Published:** 2025-09

**Authors:** Barbara Šoba, Sandra Kolar, Albin Gačnik, Manca Radež, Timotej Petrijan, Jana Rejc Marko

**Affiliations:** Author affiliations: University of Ljubljana, Ljubljana, Slovenia (B. Šoba, M. Radež); University Medical Centre Maribor, Maribor, Slovenia (S. Kolar, A. Gačnik, T. Petrijan, J. Rejc Marko)

**Keywords:** *Taenia crassiceps*, parasites, neurocysticercosis, meningitis, Slovenia

## Abstract

We present a case of subarachnoid neurocysticercosis caused by *Taenia crassiceps* in an elderly woman in Slovenia with no underlying disease or immunosuppressive treatment. The parasite was identified by 12S rDNA PCR and sequencing. Despite prolonged therapy with albendazole and praziquantel, the disease recurred after treatment was discontinued.

Human neurocysticercosis is a severe infection of the central nervous system, generally caused by larvae of the tapeworm *Taenia solium* and, rarely, by other *Taenia* species, such as *T. crassiceps*. So far, 2 cases of *T. crassiceps* neurocysticercosis have been reported in humans (*1*,*2*).

The adult *T. crassiceps* is an intestinal parasite of carnivores, mainly foxes; small mammals, such as rodents, serve as natural intermediate hosts for cyst-like larvae that proliferate by budding in their body cavities or subcutaneous tissues, leading to massive infections. Humans can become accidental intermediate hosts by ingesting parasite eggs excreted in the definitive hosts’ feces or by contamination of open wounds with eggs, as suspected in subcutaneous infections (*3*). In addition to neural and subcutaneous infections, infestation of eyes, muscle tissue, and tendons has been reported in humans (*1*,*3*). We describe a case of *T. crassiceps* infection in an elderly patient with meningitis and progressive deterioration of neurologic symptoms diagnosed by a combination of serologic and molecular methods.

Neurologic symptoms developed in a 74-year-old woman from northeastern Slovenia with no underlying diseases in December 2022. Symptoms worsened and led to gait ataxia; tetraparesis, which was markedly left-sided; urinary incontinence; and cognitive decline within a year.

Lumbar puncture (LP) performed in May 2023 confirmed aseptic meningitis. Cerebrospinal fluid (CSF) showed elevated protein levels (0.72 g/L; reference range 0.15–0.45 g/L). Glucose (2.8 mmol/L; reference range 2.5–3.9 mmol/L) and glucose ratio between CSF and serum (0.44; reference >0.31) were unremarkable. Pleocytosis was present with a total leukocyte count of 108 × 10^6^ (reference <5 × 10^6^) cells/L. Analysis of CSF sediment revealed 1% neutrophils, 75% lymphocytes and 3% plasma cells, 9% monocytes, and 12% eosinophils. Intrathecal synthesis of IgG (163.9 mg/L), IgM (5.0 mg/L), and IgA (5.7 mg/L) was confirmed. Results of blood tests, including a differential blood count, were unremarkable.

Magnetic resonance imaging (MRI) of the brain revealed an enlarged ventricular system that was more pronounced on the right side without changes in the brain parenchyma. Follow-up LPs confirmed the persistence of pleocytosis in the CNS. Extensive microbiological analyses of CSF and blood samples for infectious agents (Appendix) and tests for autoimmune and paraneoplastic encephalitis were repeatedly negative, with the exception of serologic testing of blood and CSF samples for *T. solium* IgG, which was equivocal (Figure 1). Because this result was suspicious for neurocysticercosis, we tested CSF using cestode-specific PCR amplifying the mitochondrial 12S rRNA gene (*4*); the result was positive. After sequencing and BLAST analysis (https://blast.ncbi.nlm.nih.gov/Blast.cgi) of the 242-bp amplicon obtained (Gen Bank accession no. PQ764695), the sequence showed 100% homology with *T. crassiceps*.

**Figure 1:**
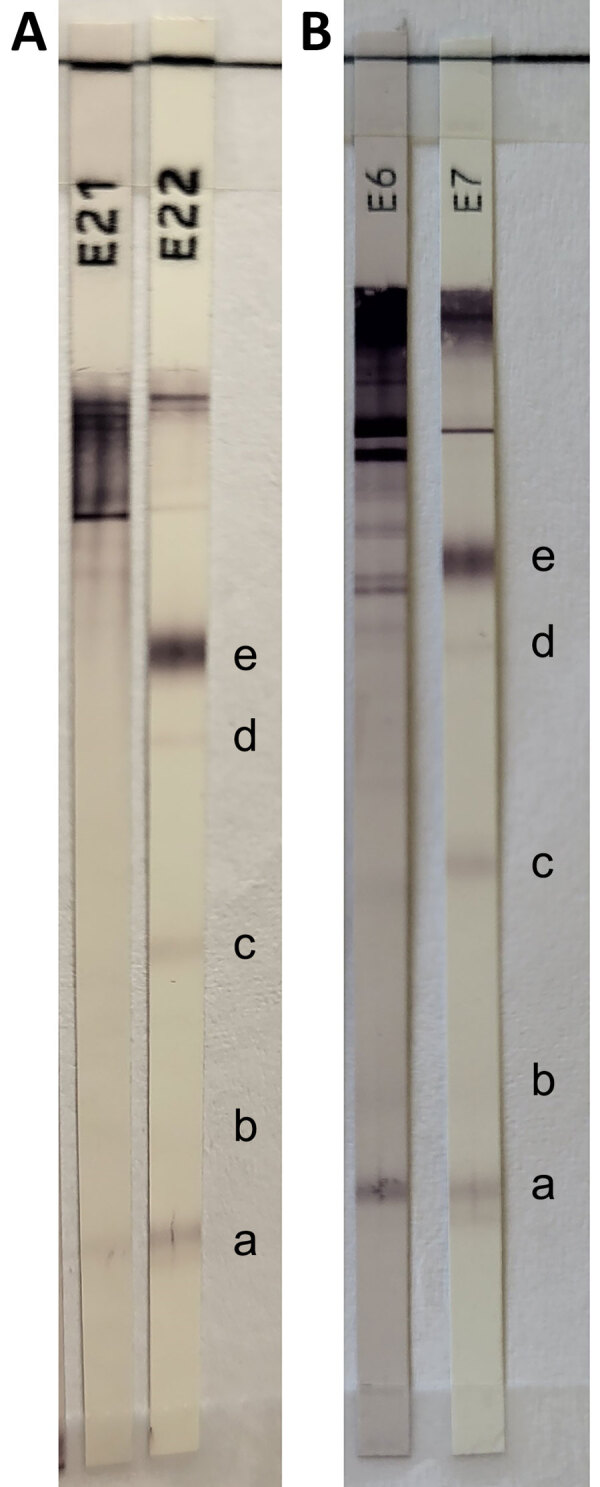
Equivocal results of Cysticercosis Western blot IgG (LDBIO Diagnostics, https://ldbiodiagnostics.com) assay in study of subarachnoid neurocysticercosis caused by larval-stage *Taenia crassiceps* tapeworm, Slovenia. Results of patient’s serum (A) and cerebrospinal fluid (B) samples show 6–8 kDa band and a weak 12 kDa band. Cysticercosis-specific bands are 6–8 kDa (a), 12 kDa (b), 23–26 kDa (c), 39 kDa (d), and 50–55 kDa (e). The presence of >2 well-defined bands among the 5 mentioned is indicative of neurocysticercosis. E21, patient’s serum; E6, patient’s cerebrospinal fluid; E7 and E22, positive controls.

MRI of the brain with contrast performed in March 2024 showed changes consistent with a subarachnoid form of neurocysticercosis (Figure 2). Results of investigations to identify additional foci of cysticercosis, including MRI of the spinal cord, were unremarkable.

**Figure 2:**
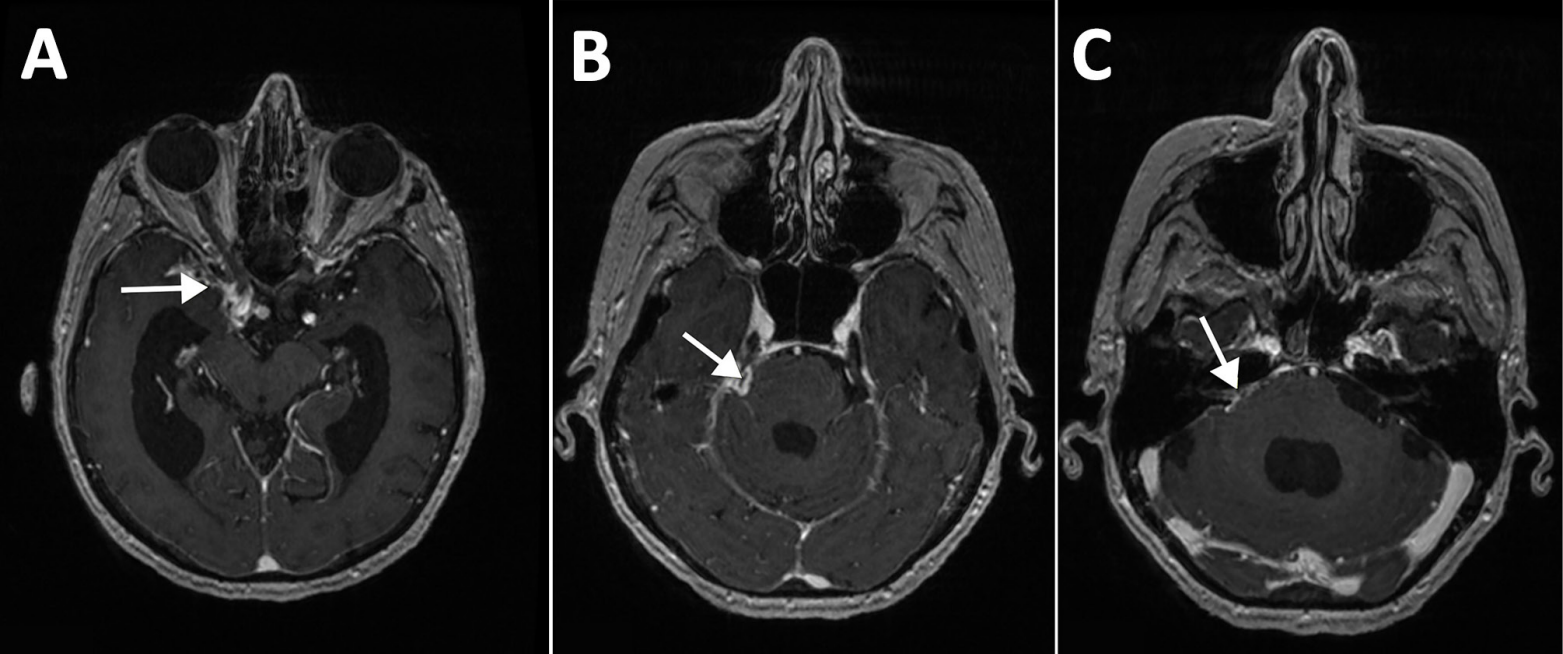
Magnetic resonance imaging of the brain in study of subarachnoid neurocysticercosis caused by larval-stage *Taenia crassiceps* tapeworm, Slovenia. Imaging shows pathological signal enhancement at the site of inflammation after contrast administration (white arrows) and a dilated ventricular system (hydrocephalus) as a result of impaired cerebrospinal fluid drainage in the right basal cisterns (A), right parapontine basal cistern (B), and right pontocerebellar angle (C).

We initiated dual therapy for neurocysticercosis with albendazole (800 mg/d) and praziquantel (2,400 mg/d), along with dexamethasone (6 mg/d) to prevent inflammation, in April 2024. Dexamethasone was administered for 4 weeks and praziquantel with albendazole for 85 days. After 85 days of therapy, subsequent CSF PCRs were negative. Praziquantel was discontinued, and treatment with albendazole was continued for a further 80 days. An LP performed 10 days after discontinuation of treatment showed no signs of meningitis. One month later, eosinophilic meningitis was again confirmed with a total leukocyte count of 20 × 10^6^ cells/L in CSF, of which 27% were eosinophils in the sediment, and PCR was again positive. The patient was restarted on dexamethasone, albendazole, and praziquantel for 2 weeks in November 2024, after which she continued treatment with albendazole. At least 1 year of albendazole treatment was planned and, in case of recurrence, lifelong therapy.

The patient’s cognitive status improved substantially after treatment, but spastic tetraparesis did not. She was no longer able to care for herself and moved into a nursing home.

Human *T. crassiceps* infections are rare; 16 cases have been reported during 1973–2023, mostly in immunocompromised but also in immunocompetent persons (*1*–*3*,*5*–*9*). Although our patient had no underlying conditions, her age might have led to immunosenescence, increasing her susceptibility to infection.

Laboratory diagnosis of *T. crassiceps* cysticercosis is challenging, especially when no clinical material is available for parasitological and pathological evaluation, as in our patient who had subarachnoid neurocysticercosis. The cystic appearance of the larvae was not visible on MRI, which differs from 2 other described cases of the parenchymal form of the disease (*1*,*2*). Equivocal or weak positive results of serologic tests for other helminthiases might indicate a possible infection (*1*,*8*,*9*). In fact, the initial suspicion of cysticercosis in this patient arose from equivocal blood and CSF *T. solium* serologic testing.

The source of this patient’s infection is unknown, but she owned a dog, as did several other reported case-patients (*1*,*5*,*7*,*8*). Increased recreational activity in wildlife areas raises risk for *T. crassiceps* infection in domestic carnivores, making regular canid deworming essential to prevent infections in humans.

Appendix Additional information about subarachnoid neurocysticercosis caused by larval-stage *Taenia crassiceps* tapeworm, Slovenia.

## References

[1] Ntoukas V, Tappe D, Pfütze D, Simon M, Holzmann T. Cerebellar cysticercosis caused by larval *Taenia crassiceps* tapeworm in immunocompetent woman, Germany. Emerg Infect Dis. 2013;19:2008–11. 10.3201/eid1912.130284 24274258 PMC3840866

[2] Floß N, Dolff S, Junker A, Blau T, Rauschenbach L, Sure U, et al. Cerebral *Taenia crassiceps* larvae infection in a 71-year-old immunocompetent male. Infection. 2023;51:277–81. 10.1007/s15010-022-01912-w 36083404 PMC9879806

[3] Deplazes P, Eichenberger RM, Grimm F. Wildlife-transmitted *Taenia* and *Versteria* cysticercosis and coenurosis in humans and other primates. Int J Parasitol Parasites Wildl. 2019;9:342–58. 10.1016/j.ijppaw.2019.03.013 31338294 PMC6626850

[4] Roelfsema JH, Nozari N, Pinelli E, Kortbeek LM. Novel PCRs for differential diagnosis of cestodes. Exp Parasitol. 2016;161:20–6. 10.1016/j.exppara.2015.12.010 26704662

[5] Goesseringer N, Lindenblatt N, Mihic-Probst D, Grimm F, Giovanoli P. *Taenia crassiceps* upper limb fasciitis in a patient with untreated acquired immunodeficiency syndrome and chronic hepatitis C infection—the role of surgical debridement. J Plast Reconstr Aesthet Surg. 2011;64:e174–6. 10.1016/j.bjps.2011.02.011 21385665

[6] Heldwein K, Biedermann HG, Hamperl WD, Bretzel G, Löscher T, Laregina D, et al. Subcutaneous *Taenia crassiceps* infection in a patient with non-Hodgkin’s lymphoma. Am J Trop Med Hyg. 2006;75:108–11. 10.4269/ajtmh.2006.75.108 16837716

[7] Roesel C, Welter S, Stamatis G, Theegarten D, Tappe D. Management of a chest-wall soft-tissue tumor caused by an infection with the larval tapeworm pathogen *Taenia crassiceps.* Am J Trop Med Hyg. 2014;91:541–3. 10.4269/ajtmh.14-0185 24914004 PMC4155556

[8] Schmid S, Grimm F, Huber M, Beck B, Custer P, Bode B. *Taenia crassiceps* infection—an unusual presentation of a tapeworm diagnosed by FNA cytology and PCR. Cytopathology. 2014;25:340–1. 10.1111/cyt.12092 24102803

[9] Tappe D, Berkholz J, Mahlke U, Lobeck H, Nagel T, Haeupler A, et al. Molecular identification of zoonotic tissue-invasive tapeworm larvae other than *Taenia solium* in suspected human cysticercosis cases. J Clin Microbiol. 2016;54:172–4. 10.1128/JCM.02171-15 26491175 PMC4702721

