# Supplementary material for: Subarachnoid Neurocysticercosis Caused by Larval-Stage Taenia crassiceps Tapeworm, Slovenia
Source: Emerg Infect Dis. 2025 Sep;31(9):1865–8. doi: 10.3201/eid3109.250014 (PMC12407208; doi:10.3201/eid3109.250014)
Supplement: Appendix — Additional information about subarachnoid neurocysticercosis caused by larval-stage Taenia crassiceps tapeworm, Slovenia. [file 25-0014-Techapp-s1.pdf]

Article DOI: https://doi.org/10.3201/eid3109.250014

EID cannot ensure accessibility for supplementary materials supplied by authors. Readers who have difficulty accessing supplementary content should contact the authors for assistance.

Subarachnoid Neurocysticercosis Caused by Larval-Stage *Taenia crassiceps* Tapeworm, Slovenia

Appendix

Information on the microbiological analyses of the patient's cerebrospinal fluid (CSF) and blood samples

The patient's samples were tested from May 2023 to March 2024 for *Mycobacterium* spp. culture (CSF), *Micobacterium tuberculosis* – PCR (CFS), bacterial culture (CSF), *Borelia burgdorferi* sensu lato – IgM, IgG (blood, CSF), *Treponema pallidum* – antibodies (blood), *Tropheryma whipplei* – PCR (CSF, feces), *Listeria monocytogenes* – PCR (CSF), *Cryptococcus neoformans* – antigen (CSF), *Noccardia* sp. culture (CSF), Human immunodeficiency virus – screening (blood), Herpes simplex virus 1 – PCR (CSF), Herpes simplex virus 2 – PCR (CSF), Varicella zoster virus – PCR (CSF), Epstein-Barr virus – PCR (CSF), Cytomegalovirus – PCR (CSF), Human herpesvirus 6 – PCR (CSF), enteroviruses – RT-PCR (CSF), Human parechovirus – RT-PCR (CSF), Human T-cell lymphotropic virus – antibodies (blood), *Toxoplasma gondii* – PCR (CSF), *Brucella* sp. – IgM, IgG (blood), *Coxiella burnetii* – IgM, IgG (blood), *Francisella tularensis* – IgM, IgG (blood), *Leptospira* spp. – antibodies (blood) and *Anaplasma phagocytophilum* – IgG (blood). All tests were negative.
